# Evaluation of Annual Physical Health Monitoring of Inpatients at a Rehabilitation Psychiatry Unit

**DOI:** 10.1192/bjo.2025.10504

**Published:** 2025-06-20

**Authors:** Heather McAdam, Sarah Morgan, John Summers, Ciara Kelly

**Affiliations:** 1 University of Glasgow, Glasgow, United Kingdom; 2 NHS Greater Glasgow and Clyde, Glasgow, United Kingdom

## Abstract

**Aims:** Individuals with severe mental illness (SMI) are at significantly higher risk of physical health comorbidities compared with the general population. Factors such as long-term antipsychotic use, lifestyle choices, and reduced healthcare engagement contribute to this increased risk. Comprehensive annual physical health checks are recommended to identify and manage these risks. This study aimed to evaluate and improve the process of conducting annual physical health checks for patients with SMI in a Glasgow psychiatric rehabilitation unit, focusing on identifying risk factors, promoting a multidisciplinary team (MDT) approach, and ensuring timely follow-up of outstanding health concerns.

**Methods:** National guidelines from the National Institute for Health and Care Excellence (NICE), the National Institute for Health and Care Research (NIHR), and NHS Scotland were reviewed to establish key standards for physical health monitoring in psychiatric rehabilitation. A structured audit tool was developed covering systemic and lifestyle reviews, physical examinations, medication monitoring, external specialty input and general health screening. Annual health reports and clinical notes were retrospectively reviewed for 30 inpatients with a minimum one-year admission between November 2023 and October 2024. Based on audit findings, a new structured health check template and an improved MDT handover protocol were implemented before re-auditing their next review.

**Results:** Twenty-eight patients agreed to be reviewed, with 25 assessed using the old template and 15 so far with the new template. The proportion of patients receiving their health check within 12 months increased from 28% (7/25) to 73.3% (11/15). Physical examinations were documented in 96% (24/25) of previous reviews, with action-oriented comments in 40% (10/25). Following the introduction of the new template, documentation increased to 100%, with 53.3% (8/15) of cases including actionable comments. Systemic enquiry documentation improved from 92% (23/25) to 100%, with action-orientated comments rising from 36% (9/25) to 73.3% (11/15). Health screening documentation improved from 60% (15/25) to 100%, with 60% (9/15) requiring action. Diabetes risk was previously recorded in only 8% (2/25) of cases but increased to 100%, with 75% (10/15) prompting action. Previously, 60% (15/25) of outstanding health concerns were discussed within the MDT, whereas 86.6% (13/15) were formally addressed post-implementation.

**Conclusion:** This study highlights the effectiveness of a structured template in improving the quality and consistency of annual physical health checks in psychiatric rehabilitation. The new template enhanced documentation, facilitated multidisciplinary discussions, and improved identification of health risks. Strengthening MDT handover processes ensures timely follow-up, reinforcing the importance of structured, standardised approaches in psychiatric physical healthcare.

